# Underwater imaging dataset in a very shallow water environment of Pramuka Island, Seribu Island District, Indonesia

**DOI:** 10.1016/j.dib.2023.109448

**Published:** 2023-07-26

**Authors:** Fickrie Muhammad, Poerbandono, Harald Sternberg, Eka Djunarsjah, Hasanuddin Z. Abidin

**Affiliations:** a Hydrography Research Group, Faculty of Earth Sciences and Technology, Bandung Institute of Technology, Indonesia; b Department of Hydrography and Geodesy, HafenCity University Hamburg, Germany; c Geodesy Research Group, Faculty of Earth Sciences and Technology, Bandung Institute of Technology, Indonesia

**Keywords:** Computer vision, Ar-Track marker tracking, Underwater imagery, Natural seabed, Coral reef environment

## Abstract

In this article, we present a dataset of underwater videos captured through manual dives in a complex and unstructured seabed area dominated by harbor structures and coral reefs. The area is shallow (0.5 – 7.0 m depth) with an enclosed embayment for the harbor area, offering protection from ocean currents and waves. The coral reef area is located in a more open ocean sloping gently toward the deeper seafloor, leading to a more pronounced rolling shutter effect and camera motion.

The dataset was collected using a GoPro Hero 10 camera, employing a standard wide lens with a horizontal field of view (FoV) of 109° and 768 × 432 image resolution. The camera is also equipped with an Inertial Measurement Unit (IMU) sensor, comprising a 200 Hz frequency accelerometer and gyroscope. During underwater deployment, the camera is protected with a 5 mm thick flat glass panel. This camera setting hence creates three medium layers of water-glass-air leading to additional refraction distortion.

To address the refraction distortion, the dataset has been subject to pre-calibration utilizing flat refractive geometry found in the Pinax camera model. The Pinax camera model for the underwater imagery is calculated by combining the aspects of pinhole calibration parameters with axial camera projection. The main aim of the dataset collection is to facilitate the testing and evaluation of underwater imaging algorithms that are used in underwater robotics, such as computer vision, photogrammetry, and Simultaneous Localization and Mapping (SLAM).

**Specifications Table**

**Table:** 

Subject	Computer Vision and Pattern Recognition
Specific subject area	Underwater imaging: Underwater imaging is a critical component of vision-based navigation and mapping in the field of underwater robotics, commonly employed in computer vision.
Type of data	1. Rosbag file; video file recorded as a Robot Operating System (ROS) bag file format. 2. Media in Low Resolution Video (LRV) file format.
How the data were acquired	The dataset has been acquired through manual dive in a shallow water environment, employing a GoPro Hero 10 camera. The camera is equipped with a standard wide lens possessing a field of view (FoV) of 109° and 768 × 432 image resolution. The camera is also equipped with Inertial Measurement Unit (IMU) sensor with 200 Hz frequency rate for gyroscope and accelerometer. The camera is protected with flat panel glass housing with 5 mm thickness generating three distinct medium layers of water-glass-air. The calibration dataset records in both underwater and in-air environments are also available to facilitate the calibration of the camera.
Data format	1. Low Resolution Video (LRV) file format contains raw video footage and motion metadata captured from GoPro 10. The metadata includes IMU telemetry data from accelerometer and gyroscope measurement. 2. Rosbag file format recorded with ROS. The rosbag file dataset contains corrected image ros topic with refraction adjustment from Pinax camera model algorithm and ground-truth recording from Ar-Track Marker detection.
Description of data collection	The raw video footages were pre-processed by using ROS ecosystem version Noetic in Ubuntu 20.04. The pre-processing includes ground truth detection using Ar-Track marker algorithm and correction map application using Pinax camera model algorithm. The correction map is used for initializing automatic camera calibration and refraction adjustment. The resulting pre-processed video footages are then saved as the rosbag file.
Data source location	Institut Teknologi Bandung, Indonesia Pramuka Island Seribu Island District Indonesia −5.7432, 106.61345
Data accessibility	Repository name: FigshareData identification number:1. Small harbor pond bay: 10.6084/m9.figshare.227699122. Near shore coral reef environment: 10.6084/m9.figshare.22886957 Direct URL to data: 1. Small harbor pond bay: https://figshare.com/articles/media/Underwater_Imaging_Dataset_at_Seribu_Island_District_Indonesia/22769912 2. Near shore coral reef environment: https://figshare.com/articles/media/Underwater_Imaging_Dataset_in_Near_Shore_Coral_Reef_Environments_at_Pramuka_Island_Seribu_Island_District_Indonesia/22886957 3. Modified Pinax camera model algorithm for GoPro 10 camera: https://github.com/fickrie67/pinax-camera-model.git The present dataset encompasses low-resolution video (LRV) format files, which are readily accessible and available for immediate utilization. Additionally, the rosbag file format included in the dataset can be played through the utilization of the ROS (Robot Operating System).

## Value of the Data

• The underwater imagery dataset has been collected through manual dives in a complex and unstructured seabed, thus providing valuable research material for researchers interested in investigating computer vision applications within real-world underwater environments.
• The dataset included within these files has been pre-calibrated using the combination Pinhole-Axial (Pinax) camera projection model algorithm, which can be used to investigate the multilayer geometry and refraction distortion effect in computer vision for underwater environments.
• The dataset is also provided by the ground truth measurement from Ar-Track marker tracking to assess the accuracy of any vision-based navigation algorithm robustness.
• Additionally, the raw dataset also stored the camera motion from IMU measurement that can be used to assess the robustness of feature detection in computer vision.

## 1 Objective

The main objective behind the generation of this dataset is to provide an open, accessible, and comprehensive resource from authentic underwater environments to conduct computer vision research and studies. The dataset offers an opportunity to investigate the effects of the underwater environment captured in the camera scene due to the density differences in the medium. The medium differences will affect the ray light propagation and cause the error point projection in the camera axis [1]. The structure of the dataset facilitates in-depth exploration in the rosbag file curated from robot operating system (ROS) tools, which contains raw video footage, adjusted video footage, and ground truth data obtained through Ar-Track marker detection.

The adjusted video footage is specifically addressing the refraction effect utilizing the Pinhole-Axial (Pinax) camera model algorithm. The Pinax camera model constructs the ray transmission through medium layers with flat panel glass geometry from [1], creating an image rectification correction map. This aspect enables researchers to examine the impact of lens and refraction distortion caused by the underwater medium and assess the effectiveness of the applied algorithm. Furthermore, the dataset includes camera calibration footage taken in-air and underwater environments, enabling the study of various calibration algorithms and the potential development of new camera calibration models. The suitability of the dataset for 3D seabed reconstruction and benthic habitat mapping is reinforced by the inclusion of data recorded in a closed-loop track. Lastly, the dataset serves as a valuable resource for analyzing underwater vision-based mapping and navigation using computer vision and ROS.

## 2 Data Description

**Fig. 1:**
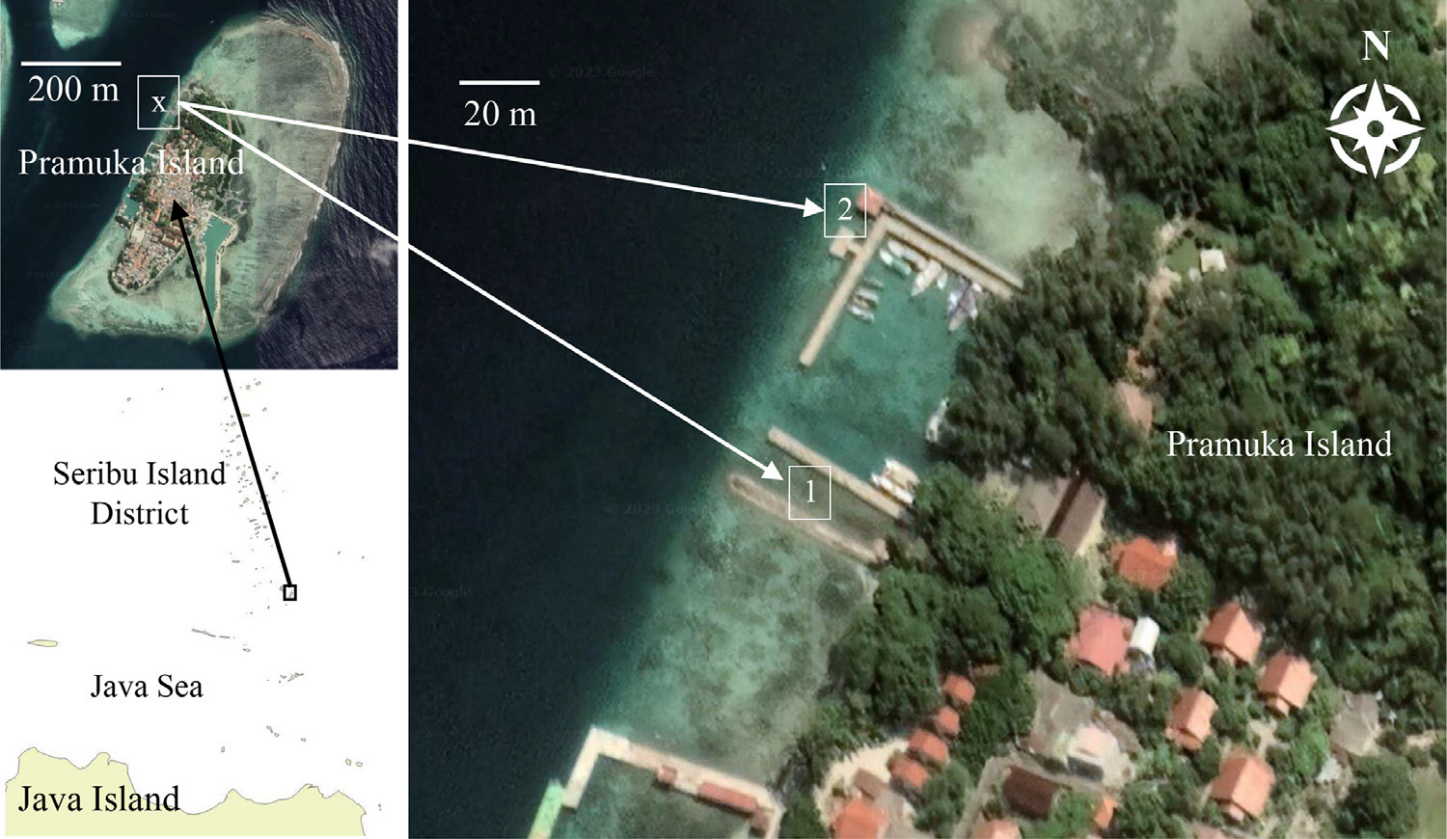
The dive location for dataset acquisition with (1) the small harbor pond area and (2) the near-shore coral reef environment area.

The dataset is collected through manual (free) dive series in two different areas at Pramuka Island, Seribu Island District, Indonesia as shown in Fig. 1. These areas are located near the shore and have shallow water depths ranging from 0.5 to 7.0 m. Area 1 (see Fig. 1) is located in a small harbor pond with an approximate depth of 0.5 m. It is characterized by a sandy seabed and occasionally covered by a harbor platform, which is useful for feature tracking. Area 2 (see Fig. 1) is situated in a more exposed location and has depths of up to approximately 7.0 m. In this area, the disturbance caused by ocean currents and waves is more prevailing, leading to rougher camera motion. Area 2 is characterized by the presence of coral reefs, contributing to more complex seabed features. The Secchi disk reading in Pramuka waters is ranging between 7.0 and 11.0 m, indicating a clear water environment around the survey areas.

The data structure encompasses two distinct seabed types, each of which offers significant potential for further utilization. Within the repository, the data is organized into two primary categories: raw media footage in Low-Resolution Video (LRV) file format and a rosbag file format, as outlined in Table 1. The LRV file contains additional metadata collected from the GoPro Inertial Measurement Unit (IMU), encompassing measurements from the accelerometer and gyroscope sensors. To extract the telemetry data, a telemetry extraction tool such as the GoPro Metadata Format (GPMF) parser from GoPro [2] can be employed. This data is valuable for visualizing the camera's motion and rotation throughout the diving operation. Furthermore, these files can facilitate an investigation of how the camera's general motion underwater impacts the robustness of computer vision algorithms.

**Table 1:** List of raw data (in LRV format) and processed data (in rosbag file format).

Name	Type	Video length (seconds)	Description
GL010221.LRV	Video footage (LRV)	61	In-air calibration paper detection
GL010221_all.bag	Rosbag file		
GL010106.LRV	Video footage (LRV)	54	Underwater calibration paper detection
GL010106_all.bag	Rosbag file		
GL010110.LRV	Video footage (LRV)	37	Small-scale harbor pond
GL010110_all_marker.bag	Rosbag file		
GL010111.LRV	Video footage (LRV)	94	Medium-scale harbor pond
GL010111_all_marker.bag	Rosbag file		
GL010162.LRV	Video footage (LRV)	125	Coral reef environment 1
GL010162_all.bag	Rosbag file		
GL010163.LRV	Video footage (LRV)	172	Coral reef environment 2
GL010163_all.bag	Rosbag file		

**Fig. 2:**
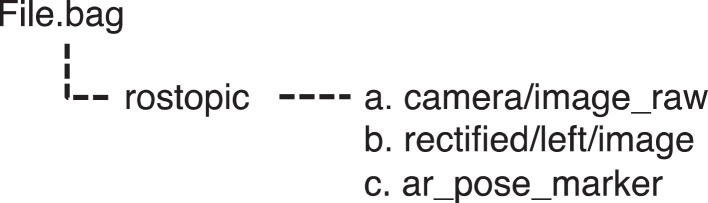
Structure of rosbag file: (a) The raw footage, (b) The post refraction adjusted with Pinax camera model, and (c) The camera position from Ar-Track marker tracking.

The ROS tools facilitate the video recording and curation in the rosbag file format. The rosbag file contains three fundamental image topics, as illustrated in Fig. 2. Specifically, the raw media footage and the pre-processed footage that has undergone post-refraction adjustment with a modified Pinax camera model for Ubuntu 20.04 with OpenCV version 4 [3] are included within the rosbag file. They are stored as camera/image_raw and rectified/left/image, respectively. This organization allows researchers to directly compare and analyze both sets of data. Additionally, the rosbag file contains ground-truth data represented in local metric Ar-Track coordinates. This coordinates system is obtained through the detection of Ar-Track markers by [4] with sub-millimeter reprojection error. This ground-truth data serves as a valuable reference for precise analysis and evaluation.

**Fig. 3:**
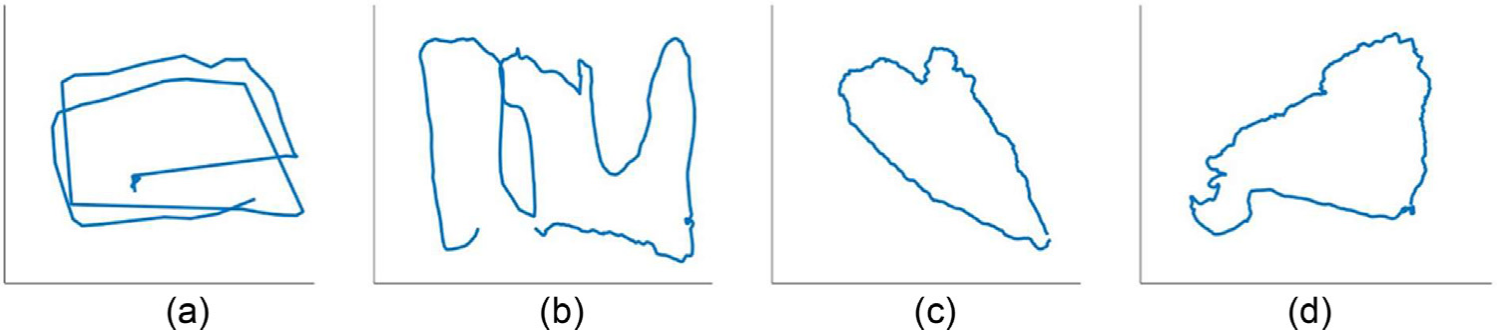
The trajectory of camera movement from the diving series of (a) small harbor pond (b) medium harbor pond (c) coral reef environment 1 (d) coral reef environment 2.

The dive series is carried out in the close-loop track in random motion which is beneficial to test the close-loop algorithm in SLAM, as shown in Fig. 3. The first and second dataset involves camera calibration footage obtained from multiview calibration paper detection from in-air and underwater recordings. The third dataset consists of footage recorded in a small harbor pond (circa 2 × 2 m covered in 37 s) with full support for closed-loop operations and detection of all Ar-Track markers. In the fourth dataset, the footage is acquired in a medium-sized harbor pond (circa 5 × 2 m covered in 94 s), where Ar-Track marker detection is conducted solely at the beginning and end of the track. Finally, the fifth and sixth dataset involves footage captured in a coral reef environment without the utilization of Ar-Track marker detection. The sixth dataset exhibits greater coverage of the coral transition zone, extending from the reef flat to the deeper reef crest. This expanded range of depths (approximately up to 7 m) and increased track length (approximately 8 × 8 m covered in 172 s) offer more seabed feature variations compared to the fifth dataset. The fifth dataset, on the other hand, covers a smaller area (circa 5 × 8 m) in a shorter video duration of 125 s. The increased coverage and variations in the sixth dataset are beneficial for testing the visual range limitations in computer vision.

## 3 Experimental Design and Data Acquisition Procedure

**Fig. 4:**
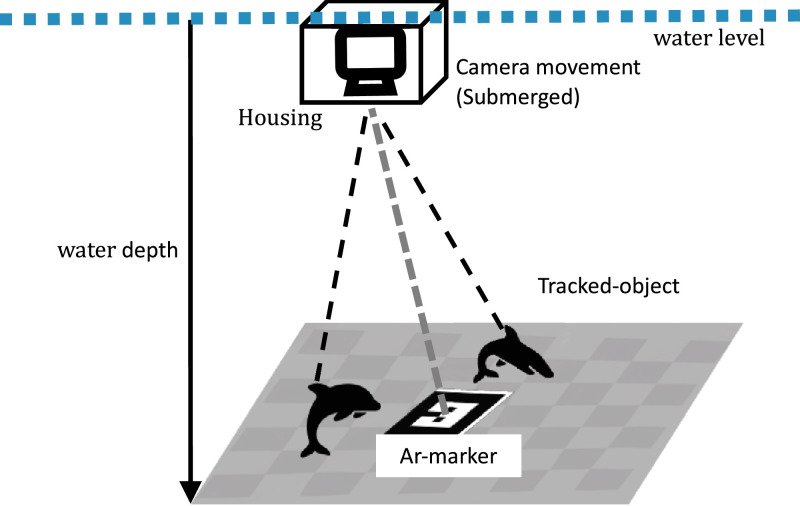
Illustration of data acquisition.

An illustration representing the process of data acquisition involving the manual dive movement of a camera submerged underwater in a complex and unstructured seabed can be seen in Fig. 4. The video recording is initiated with GoPro 10 camera that is robust in underwater environments and has wide coverage with 109°FoV. The camera is set to take video in 30 Frames per Second (FPS) which is a standard FPS to be used in feature detection algorithms found in computer vision. The camera is also integrated with an IMU sensor with a 200 Hz frequency recording rate to represent the camera motion. Enclosed in a sealed 5 mm thick flat panel glass housing, the camera is manually maneuvered (by hand) in the water column to capture the desired objects on the seabed. This method allows for the collection of data in a controlled manner, ensuring the recording of specific underwater scenes and conditions of interest, thus providing valuable research material for researchers interested in investigating computer vision applications within real-world underwater environments. Additionally, researchers interested in studying the underwater camera model and its calibration effect can use pre-processed video files stored in rosbag file format.

The marker coordinate system derived from Ar-Track marker detection facilitates the accurate metric localization of camera movements underwater [4]. In this dataset, an Ar-Track marker with dimensions of 4,8 cm is utilized, and it must be correctly measured before executing the Ar-Track marker detection algorithm. The accurate specification of the Ar-Track marker dimensions ensures precise measurements during the Ar-Track marker localization process, contributing to the overall high accuracy of the localization results.

### 3.1 Calibration footage

**Fig. 5:**
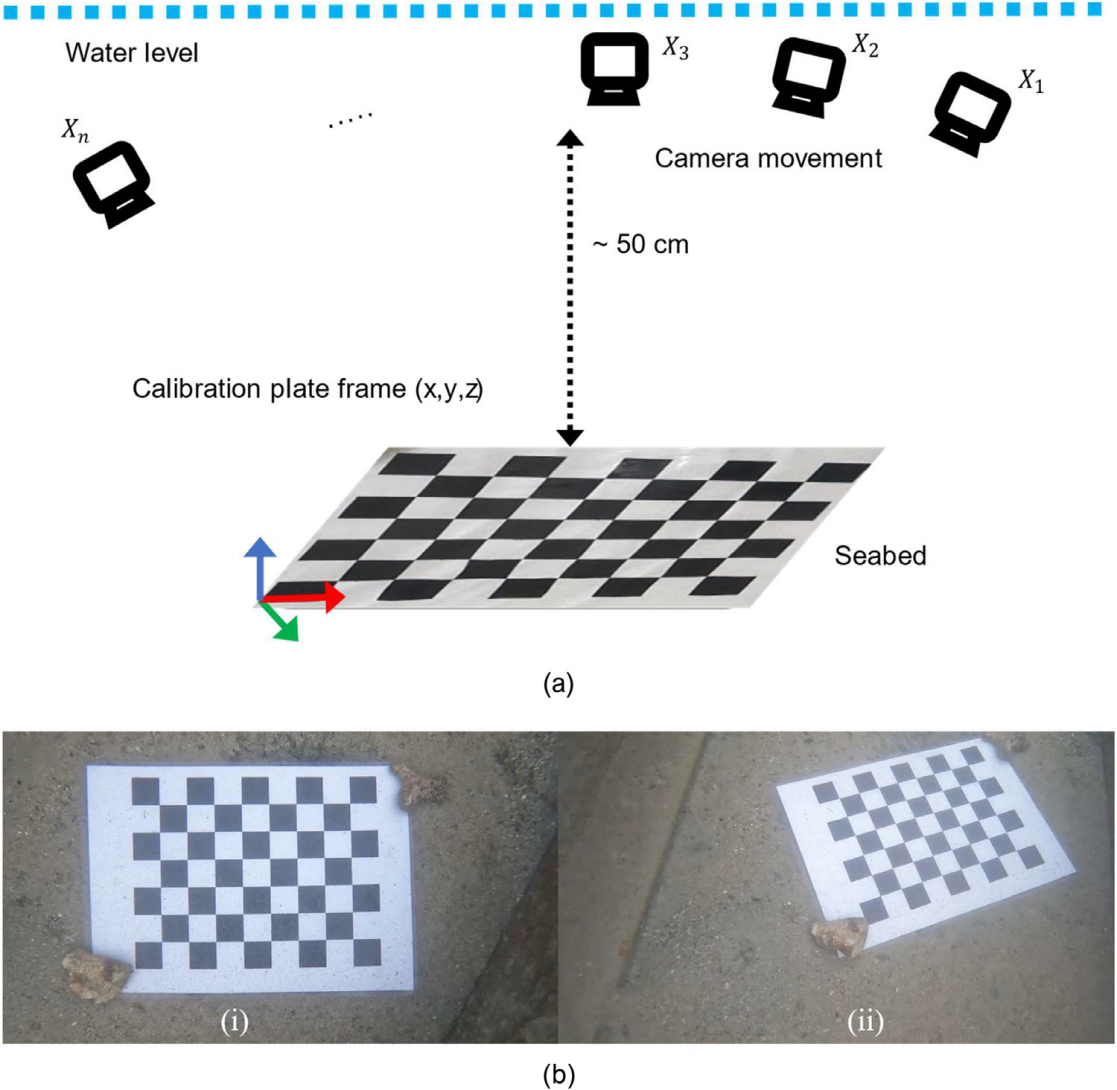
Camera calibration procedure: (a) Illustration of camera movement above checkerboard calibration pattern. (b) Typical example of the image sequence in the small harbor pond bay for camera calibration underwater: (i) vertical, (ii) oblique.

The use of camera sensors shall address the lens distortion, namely radial and tangential distortion prior to any further use in computer vision [5]. In an underwater environment, the refraction effect shall also be taken into account which may cause large error propagation over time. To mitigate these effects, the conventional method of standard camera calibration shall be applied. Multiview camera calibration techniques have demonstrated suitability in the context of underwater refraction adjustment. However, it is important to note that this technique requires the processing of a substantial number of images to accurately compute the distortion parameters from various angles and positions [6]. The calibration process entails using a known set of control points, typically obtained from a calibration pattern or target with known coordinate origins and dimensions. One commonly used calibration pattern is a checkerboard pattern placed on a rigid surface or vice versa. To facilitate the camera calibration, a feature detection algorithm is employed to detect and locate the checkerboard pattern within the camera's field of view [7]. Fig. 5 shows the target is captured by the camera from multiple angles and positions, enabling the collection of the necessary translation and rotation parameters required for subsequent intrinsic and extrinsic parameter calculations.

### 3.2 Refraction adjustment

**Fig. 6:**
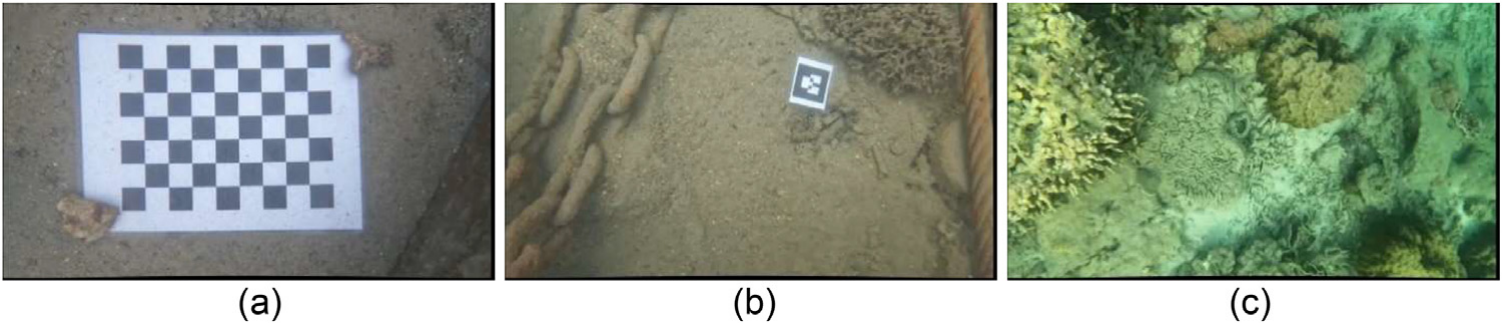
Example of image footage with refraction adjustment in: (a) underwater calibration paper detection at a depth of 0.5 m, (b) small harbor pond bay with Ar-Track marker detection at a depth of 0.5 m (c) coral reef environment at an approximate depth of 2.0 to 3.0 m.

Fig. 6 illustrates image footage examples that demonstrate the visual effects of the distortion and refraction correction achieved through the utilization of the Pinax camera model algorithm. The Pinax camera model represents the 3D seabed point projection using the Pinhole camera model combined with the flat refractive geometry to accumulate the axial point projection in the different medium layers [3]. The Pinax calibration model builds upon the standard camera calibration process, incorporating an additional scale adjustment to account for refraction effects introduced by the transition between different mediums, such as water, glass, and air. These refraction-induced scale adjustments are considered part of the extrinsic parameters in the Pinax camera model [3]. To apply the Pinax camera model, intrinsic parameters are first required, which can be obtained through an in-air calibration process. This implies that the camera does not need to submerge underwater during calibration. In the dataset, the intrinsic parameters are calculated from the standard Pinhole camera calibration model. The intrinsic parameters used in the dataset consist of the following components: the focal length, denoted as *fx* (458.058) and *fy* (460.956); the camera principal point *cx* (387.907) and *cy* (210.587); the radial lens distortion coefficients *K1* (−0.247) and *K2* (0.0869); and the tangential lens distortion coefficients *P1* (−0.006) and *P2* (0.001).

The extraction of extrinsic parameters involves 3D seabed points projection underwater towards the glass panel and then directly projecting them onto the camera's image plane using the known intrinsic parameters. The adjusted footage incorporates the standard refraction indices of the glass of 1.492, the refraction indices of water of 1.342 estimated with a look-up table from [8], and intrinsic parameters from in-air calibration. The glass thickness is set to 5 mm, and the virtual distances of the camera axis are specified as 0.39 mm. Notably, these parameters are essential for the accurate application of the pre-processed distortion correction using the Pinax camera model algorithm.

## Ethics Statements

This data article does not involve experiments on humans or animals.

## CRediT Author Statement

**Fickrie Muhammad:** Conceptualization, methodology, data collection, data curation, Writing – original draft preparation; **Poerbandono:** Supervision, Reviewing, and editing; **Harald Sternberg:** Supervision and reviewing, Funding acquisition; **Eka Djunarsjah:** Reviewing; **Hasanuddin Z Abidin:** Reviewing.

## Declaration of Competing Interest

The authors declare that they have no known competing financial interests or personal relationships that could have appeared to influence the work reported in this paper.

## Data Availability

Underwater Imaging Dataset in Near Shore Coral Reef Environments at Pramuka Island, Seribu Island District, Indonesia (Original data) (Figshare). Underwater Imaging Dataset in Small Harbor Pond Bay at Pramuka Island, Seribu Island District, Indonesia (Original data) (Figshare).
